# Frequency and correlates of violence against patients with schizophrenia living in rural China

**DOI:** 10.1186/s12888-020-02696-9

**Published:** 2020-06-06

**Authors:** Qian-Wen Wang, Cai-Lan Hou, Shi-Bin Wang, Zhuo-Hui Huang, Ying-Hua Huang, Ji-Jie Zhang, Fu-Jun Jia

**Affiliations:** 1 The Second School of Clinical Medicine, Southern Medical University, Guangzhou, Guangdong Province China; 2 Guangdong Provincial People’s Hospital, Guangdong Academy of Medical Sciences, Guangdong Mental Health Center, Guangzhou, Guangdong Province China

**Keywords:** Schizophrenia, Victimization, QoL, Social function, Rural China

## Abstract

**Background:**

Violence against patients with schizophrenia is very common, however it is rarely studied in China, especially in primary health care institutions of rural areas. Therefore, we investigated the frequency of violence against patients with community-living schizophrenia in rural China and examined its associated factors and impact on quality of life (QoL) and social function.

**Method:**

A survey was conducted among 487 patients with schizophrenia living in rural communities. Data about violent victimization experiences in the past 6 months, demographic information, and clinical characteristics were collected by questionnaires.

**Results:**

We found that 92 (18.9%) of 487 subjects experienced at least one type of violent event in the past 6 months. Logistic regression analysis suggested that a history of conducting dangerous behaviors(OR = 1.702, *P* = 0.02, 95%CI: 1.05–2.73), higher Brief Psychiatric Rating Scale (anxiety domain) score (OR = 1.15, *P* = 0.02, 95%CI: 1.01–1.304) and lower hospitalization rates (OR = 0.89, *P* = 0.04, 95%CI: 0.81–0.99) were significantly associated with violent victimization in patients with schizophrenia. Analysis of covariance showed the victims of violence tended to have worse social function in patients with schizophrenia living in rural communities of China (*P* = 0.04).

**Conclusions:**

Individuals with schizophrenia living in rural China had a high risk of being exposed to violence and violent victimization of patients with schizophrenia had adverse consequences for social function. More attention is needed for those patients experiencing violent events, because they are simultaneously possible to conduct dangerous behaviors.

## Background

Researchers concerned about violence perpetrated by persons with severe mental illness (SMI) in decades [1]. The public also had a common perception that SMI such as schizophrenia were dangerous because they contributed to severe violence [2]. However, some studies suggested that violent victimization was found to be more widespread than perpetration among individuals with severe mental illness [1, 3]. Unfortunately, past studies did not pay much attention to violent victimization of SMI compared with dangerous behaviors by the patients in recent years [4].

A US study on patients with schizophrenia living in the community reported that 38% of the samples had been the victims over a 3-year period, 65–130% higher than the general population [5]. The one year prevalence of victimization was 16.8% in a study among patients with SMI in Taiwan, compared with 11.3% in the general population [6]. In a review, the prevalence of violent victimization in patients with SMI under treatment was 8.2% in the past four months and 35.0% in the past year [1]. Prevalence rates of violent victimization in SMI in the past year ranged from 25.3 to 35.0% compared with 2.9% in the National Crime Victimization Survey during the same period [1].

In previous investigations on violence against patients with SMI, the occurrence of violent attacks was found to be related to adverse health outcomes containing impairment of functioning and quality of life [4, 7]. Violent victimization of people with SMI was associated with younger age, alcohol or drug abuse, being a perpetrator of violence, more severe symptomatology, and homelessness etc. [7–10]

However, above results cannot be generalized to patients with SMI in China. Unlike western countries, deinstitutionalization has not been the common phenomenon in most Asian countries including China. Meanwhile, the lack of psychiatrists in rural China is very prominent. Primary care physicians provide basic medical services for a large proportion of clinically stable patients with SMI including schizophrenia in China. In addition, patients with schizophrenia living in rural communities in China are primarily cared by their relatives at home [11]. Furthermore, the characteristics like the rates of employment among patients with schizophrenia living in rural China are differed from patients living in high-income countries [12].

Although violent victimization in people with SMI had frequently occurred and high incidence of adverse consequences of victimization was documented in previous studies [8], it was not thoroughly studied in China. Researchers yielded inconclusive results with respect to the rate of victimization in urban or rural residential areas [13]. In addition, Missiry et al. found that patients with different mental illness diagnoses had different rates of violent victimization [14].

In order to eliminate heterogeneity of diseases, this study focus on the frequency and associated factors of violence against patients with schizophrenia in rural communities in China. Taken together, we designed this study (i) to explore the 6-month prevalence of violence against patients with schizophrenia living in rural communities; (ii) to analyze possible factors of being victims of violence; and (iii) to investigate the influences of violent victimization on the quality of life (QoL) and overall functioning in patients with schizophrenia.

## Methods

### Study design and participants

This was a cross-sectional study initiated by the Guangdong Mental Health Center of Guangdong Provincial People’s Hospital between November 8, 2017 and January 25, 2018. The study was conducted in rural areas around Luoding county which located at the western part and belonged to an underdeveloped area of Guangdong Province.

There were a total of 63 townships with primary health care services in Luoding county under the jurisdiction of Yunfu City [15]. Twenty-one township primary care services in Luoding county were chosen by random cluster sampling method in our study. All local community-dwelling patients with schizophrenia who had presented to primary care services were registered in the Chinese National Psychiatric Management System, in which individuals with SMI, including schizophrenia, bipolar disorder, paranoid psychosis, schizoaffective disorder, mental disorder related to epilepsy, and mental retardation with psychotic symptoms were required to enroll in this national system. We then contacted all the patients meeting inclusion criteria by telephone calls and provided a detailed interpretation about our research. If they accepted to sign the consent and agreed to continue the whole assessment, one of three trained psychiatrists made an appointment for the interview at the local primary care service. A total of 742 participants were contacted, 489 agreed to take part in the survey and two of them did not finish the interview. The response rate was 65.9% (489/742).

Patients were identified by systematic review of Chinese National Psychiatric Management System registers and fulfilled the following inclusion/exclusion criteria: (a) aged 18 years or above; (b) diagnosed as schizophrenia based on a review of medical record by ICD-10 and supplemented by a clinical interview; (c) managed by primary care physicians; (d) had ability to understand the content of the interview; and (e) absence of organic brain damage.

The study protocol was approved by the Ethics Committees of Guangdong Provincial People’s Hospital. All patients provided written informed consent.

### Measures

Patients’ basic sociodemographic and clinical characteristics including number of hospitalizations and dangerous behaviors in the last 6 months were collected by two psychiatrists using a standard form designed for the study. Data about medication prescriptions including first- and second-generation antipsychotics (FGAs and SGAs), antidepressants, anticholinergics and benzodiazepines were recorded by an electronic chart management system. Doses of antipsychotic drugs were converted into the prescribed daily dose/the defined daily dose ratio (PDD/DDD ratio) [16].

Violent victimization in the past 6 months was coherent with the definition used in previous studies [17, 18]. Violent victimization was defined as an act of sexual or physical violence experienced by a person with schizophrenia. The experience of being verbal threatened and abused was also included within the definition of violent victimization as this was considered to be a severe form of emotional violence. Violent victimization was assessed on the basis of five different episodes. Episodes of sexual assault with violence, sexual harassment with physical contacts, verbal harassment with sexual content, non-sexual physical violence and verbal threat and abuse by family members or others were included. Sexual assault with violence referred to the experience of unwelcome sexual advances involving violence; sexual harassment with physical contacts was defined as the experience of any type of unwelcome sexual behavior with body contact; verbal harassment with sexual content referred to the experience of verbal abuse with sexual content; non-sexual physical violence was defined as the experience of being subjected to physical contact (being hit, kicked, slapped, pushed, grabbed, choked, etc.) with intention to harm or injure; verbal threat and abuse referred to being sworn at, yelled, called by names or other words intended to control or hurt [18]. Participants were asked if they had experienced any types of violence in the past 6 months. If patients answered “yes” to at least one of the five aspects, they were classified as “violent victimization”. Detection of the question about violence has been used on the nurses and an Chinese version of the original instrument found to have a good inter-rater reliability [18, 19].

Patients with schizophrenia are sometimes perpetrators of violence. Therefore, in this study, we also tried to assess perpetration of dangerous behaviors among patients with schizophrenia, and they were asked if they had engaged in the following actions towards others in the past 6 months. Dangerous behavior was defined as an act of behavior (involving verbal threat, property crimes, vandalism violent crimes and violent crimes, etc.) committed by a patient with schizophrenia. Participants answered “yes” to this question will defined as “Past dangerous behaviors”.

Current smokers were assessed with the following definitions: patients who smoked at least one cigarette daily in the past month [20]. Current alcohol users were defined as drinking at least one alcoholic beverage per month during the past year [21].

Psychotic symptoms were assessed using the validated Chinese version of the Brief Psychiatric Rating Scale (BPRS): positive, negative, anxiety and tension domain [22]. The severity of depressive symptoms in the past week was assessed using the validated Chinese version of the 16-item Quick Inventory of Depressive Symptomatology (QIDS-SR) [23]. Extra-pyramidal side effects (EPS) were measured using the Simpson-Angus Scale (SAS) [24]. The Sheehan Disability Scale (SDS) assessed Functional disability with a self-report instrument that assesses work or study, social life, family life or home responsibilities with a 10-point visual analogue scale, from extreme disability (10) to no disability (0) [25]. A higher score on SDS indicates severer functional impairment. The quality of life was evaluated using the Chinese version of the WHOQOL-BREF questionnaire [26]. This is a self-report measure on QoL with physical, psychological, social and environmental domains.

The three interviewers underwent an inter-rater reliability exercise with the above-mentioned assessment tools in 20 patients with schizophrenia prior to the study. The inter-rater reliability of the rating instruments and the judgment of violent victimization yielded satisfactory agreement (>0.95).

### Statistical analysis

Data were analyzed using the Statistical Package for Social Science (SPSS 21.0). The 6 months prevalence of violent victimization was calculated for patients with schizophrenia. The difference between female and male in each type of violence was compared by chi-square tests. Comparisons in terms of demographic and clinical variables between patients experiencing violent events and those who did not were carried out by chi-square test, fisher’s exact test, t-test Mann–Whitney U test. QoL and functional impairment were compared between the above two groups using analysis of covariance (ANCOVA) after controlling for the potentially confounding effects of variables that significantly differed between the two groups in univariate analyses. Multiple logistic regression analysis with the “Backward:LR” method was used to determine the demographic and clinical variables that were independently and significantly associated with violent victimization. Violent victimization was entered as the dependent variable, while the demographic and clinical characteristics that significantly differed (P<0.05) between the two groups above were entered as independent variables. The level of significance was set at 0.05 (two-tailed).

## Result

A total of 489 patients with schizophrenia living in rural communities were eligible for the study, in which 487 met the study entry criteria, and 2 patients (1.7%) did not complete the interview. Thus 487 patients formed the study sample. None of the participants had comorbidities of psychoactive substance abuse in their clinical record.

Table 1 shows the prevalence of type of violence against patients with schizophrenia in the whole sample and separated by genders. The patients who experienced at least one of the five types of violence accounted for 18.9% (*n* = 92) of the whole sample, and 16.7% in males, 22.9% in females (X^2^ = 2.804, df = 1, *P* = 0.09). The frequency of different types of violence ranged between 0.2% and 16.0%.

**Table 1:** Prevalence of type of violence against patients with schizophrenia living in the rural community

Type of violent victimization	Total (*n* = 487)		Female (*n* = 175)		Male (*n* = 312)		Statistics		
	N	%	N	%	Male	%	X^**2**^	df	p
Violence	92	18.9	40	22.9	52	16.7	2.804	1	0.09
Sexual	19	3.9	10	5.7	9	2.9	2.39	1	0.12
Attack	1	0.2	0	0	1	0.3	–	–	1.00
Physical harassment	3	0.6	1	0.6	2	0.6	–	–	1.00
Verbal harassment	18	3.7	9	5.1	9	2.9	1.606	1	0.205
Non-Sexual	86	17.7	36	20.6	50	16.0	1.59	1	0.207
Physical violence	25	5.1	7	4.0	18	5.8	0.72	1	0.39
Verbal threat abuse	78	16.0	35	20.0	43	13.8	3.22	1	0.07

Table 2 shows the sociodemographic and clinical characteristics of victimized versus non-victimized patients with schizophrenia, and the comparison between the two groups with respect to overall functioning and QoL. Compared with the non-victimized group, patients who had been a victim of violence had a higher frequency of conducting dangerous behaviors, having severe BPRS anxiety symptoms and depressive symptoms, and less frequency of hospitalizations in the past 6 months. On the other hand, patients experiencing violent events had lower scores for environment QoL domain and worse social function. The prescribed medication for treatment of schizophrenia was not associated with violent victimization in our study.

**Table 2:** Sociodemographic and clinical characteristics of victimized versus non-victimized patients with schizophrenia

Variables	Total (*n* = 487)		Non-victimized (*n* = 395)		Victimized (*n* = 92)		Statistics		
	n	%	n	%	n	%	X^**2**^	df	p
Male gender	312	64.1	260	65.8	52	56.5	2.804	1	0.09
Married	217	44.6	169	42.8	48	52.2	2.66	1	0.103
Occupied	188	38.6	149	37.7	39	42.4	0.68	1	0.407
Living with others	449	92.2	365	92.4	84	91.3	0.12	1	0.72
Family history of psychiatric disorders disdisdisorders	110	22.6	29	22.5	21	22.8	0.004	1	0.95
Conductingdangerousbehaviors	196	40.2	149	37.7	47	51.1	5.54	1	**0.01**
Major medical conditions	27	5.5	22	5.6	5	5.4	0.003	1	0.95
Current alcohol	20	4.1	15	3.8	5	5.4	0.17	1	0.67
Currentsmoker	122	25.1	100	25.3	22	23.9	0.07	1	0.78
UseofAntipsychoticMedication							5.09	3	0.16
NoAntipsychotic	52	10.7	41	10.4	11	12.0			
FGAsonly	59	12.1	52	13.2	7	7.6			
SGAsonly	276	56.7	216	54.7	60	65.2			
FGAs+SGAs	100	20.5	86	21.8	14	15.2			
On antidepressants	19	3.9	15	3.8	4	4.3	–	–	0.76*
On benzodiazepines	75	15.4	60	15.2	15	16.3	0.07	1	0.79
On moodstablizers	108	22.2	81	20.5	27	29.3	3.38	1	0.06
On anticholinergics	200	41.1	157	39.7	43	46.7	1.507	1	0.22
	Mean	SD	Mean	SD	Mean	SD	t	df	P
Age (years)	42.36	11.89	42.34	11.94	42.46	11.75	−0.08	485	0.93
Education(years)	8.26	2.15	8.25	2.19	8.33	1.99	−0.302	485	0.76
Age of onset (years)	26.24	9.21	26.15	9.11	26.62	9.58	−0.44	485	0.65
Number of hospitalizations	3.08	2.57	3.19	2.64	2.59	2.17	2.04	485	**0.04**
BMI	24.24	4.12	24.21	4.03	24.40	4.49	−0.404	468	0.68
PDDDDDAPP Total	0.96	0.80	0.98	0.81	0.90	0.71	0.85	485	0.39
BPRS Total	24.96	6.409	24.70	6.39	26.10	6.38	−1.89	485	**0.05**
BPRS-positive	5.86	2.39	5.77	2.403	6.24	2.34	−1.68	485	0.09
BPRS-negative	5.00	2.23	5.05	2.34	4.77	1.67	1.33	485	0.18
BPRS-anxiety	5.53	1.68	5.42	1.58	6.00	2.01	−2.57	485	**0.01**
SAS Total	0.06	0.14	0.06	0.14	0.07	0.17	−0.86	485	0.39
QIDS total	5.83	3.69	5.66	3.73	6.59	3.44	−2.17	485	**0.03**
QoL Physical Domain	13.92	1.73	13.95	1.72	13.801	1.75	0.74	485	0.45
QoL Psychological Domain	12.58	1.97	12.59	2.008	12.55	1.83	0.14	485	0.88
QoL Social Relationship Domain	12.79	2.37	12.76	2.36	12.91	2.42	−0.53	485	0.59
QoL Environment Domain	12.42	1.74	12.502	1.71	12.11	1.82	1.903	485	**0.05**
SDS work	4.1	3.74	3.96	3.72	4.74	3.75	−1.79	485	0.07
SDS social	4.07	3.66	3.89	3.64	4.86	3.67	−2.28	485	**0.02**
SDS family	2.98	2.901	2.91	2.86	3.26	3.06	−1.03	485	0.302

After controlling for the above variables that significantly differed between the two groups, there was still significant difference in the social function between the two groups, (*F*(11,623) = 4.09, *P* = 0.04),but there was no difference in environment domain of QoL.

Multiple logistic regression in Table 3 revealed that a history of conducting dangerous behaviors, higher Brief Psychiatric Rating Scale (anxiety domain) score and lower hospitalization rates were independently associated with violent victimization in patients with schizophrenia, accounting for 5.3% of the variance of victimization.

**Table 3:** Independent socio-demographic correlates of violent victimization in patients with schizophrenia (non-victimized patients as the reference group)(*N* = 487)

	P	OR	95% CI
Number of hospitalizations	**0.04**	0.89	0.81–0.99
Conducting dangerous behaviors	**0.02**	1.702	1.05–2.73
BPRS anxiety	**0.02**	1.15	1.01–1.304
QIDS total	0.37	–	–

Notes: Bold values are *P*<0.05; *BPRS* Brief Psychiatric Rating Scale, *OR* odds ratio, *QIDS* Quick Inventory of Depressive Symptomatology

## Discussion

To the best of our knowledge, this was the first study to investigate self-reported rates of violent victimization over a 6-month period among community-dwelling individuals with schizophrenia in rural China. 18.9% of community-dwelling patients with schizophrenia reported at least one type of violent victimization.

Different prevalence of violent victimization was reported in previous studies, ranging from 4.3% during the past month in Melbourne concerning patients with schizophrenia and related disorders [27], and 91% during the lifetime in South Carolina in patients with mental illness [28]. If we focused on researches with the same timeframe as this study, we found two similar results which were both from America. An study among patients with schizophrenia or bipolar disorders was conducted in prison population in the USA, 24% of the male patients were victims of physical assault compared with 17.3% in the female [29]. In San Francisco-based non-profit psychosocial rehabilitation agency, 25.6% of patients with severe mental illness reported that they had been victims of a violent crime during the past 6 months [30]. However, it was also difficult to compare the rate of violent victimization to the other studies due to the heterogeneity of diagnosis or different rates of comorbid diseases and living situations. The prevalence of violent victimization ranged from 7.1 to 56% after limiting the data to studies that used the time frame of 1 year for the assessment of victimization in a review paper [4]. Different prevalence of violent victimization in different studies had multiple influential reasons. It would have the possibility that the 6 months rate of 18.9% in our study would translate to comparably a high rate of violent victimization over longer period of observation time.

Contrary to some previous studies, we found that patients having higher frequency of hospitalizations were less likely to experience violence. Liselotte D et al. [9] hypothesized that in-patients and patients living in a sheltered housing facility may benefit from training in conflict management skills that aimed to improve skills relating to interaction with other patients. Fortugno et al. [10] pointed out that the risk of the number of hospitalizations can not be generalized to other groups of patients with schizophrenia because their study involving individuals came from involuntarily admitted population who represented a group with a particularly high level of being involved in violent events, whilst only clinically stable schizophrenia patients living in rural communities were involved in our study.

In contrast to previous studies exploring risk factors for violent victimization in people with SMI or schizophrenia [8, 31], alcohol use was not associated with violent victimization in patients with schizophrenia in our study. Only 4.1% of patients with schizophrenia used alcohol, the number of alcohol users was much higher in most western countries [32]. Furthermore, the assessment of severity about alcohol use was insufficient in this study, which might limit the exploration of association between alcohol use and violence.

Logistic regression analysis in this study showed that a history of conducting dangerous behaviors, higher Brief Psychiatric Rating Scale (anxiety domain) score and lower hospitalization rates and were significantly associated with violent victimization in patients with schizophrenia.

In this study, violent victimization among patients with schizophrenia were more likely to conduct dangerous behaviors, which is in accordance with other studies. A study included patients with schizophrenia spectrum disorders showed that those exposed to violence had a higher likelihood of engaging in violent behavior [1]. The existing similar literature focused on patients with SMI, rather than a specific kind of mental illness. In summary, patients with severe mental illness who experiencing violent events were more likely to have a record of violent behaviors [3, 33–35]. This can be explained by the fact that the mental illness weakened their ability to manage conflict circumstances as well as expressing negative feelings. This possible reason leaded to higher opportunities for patients becoming a victim of violence as well as a perpetrator of dangerous behaviors [4]. Another mechanism could be that conducting dangerous behaviors may be a response to provocation or threats from others. A third explanation would be that dangerous behaviors from patients with schizophrenia provoked violence from others in a self-defense situation [34].

There was no consensus about the relationship between specific symptoms and violent victimization among severe mental illness individuals. However, Brekke et al. found no association between severity of symptoms and risk of violent victimization in schizophrenia [5]. De Mooij et al. estimated that patients with severe symptoms of disorganisation were more likely to be victimized in SMI [9]. However, our study demonstrated that patients of schizophrenia with severe anxiety symptoms had a close association with being a victim of violence. Some studies provided evidence on the specific importance of anxiety symptoms to some extent [36]. Anxiety could trigger disease relapses [37],whistle severe symptoms was associated with victimization. There were other explanations, anxiety was associated with facial emotion recognition deficits [38]. Meanwhile, false interpretation of facial may contribute to inappropriate social behavior, including reacting aggressively even in innocuous social situations that will increase the possibility of being victimized [39].

As would be expected, the presence of violent victimization was significantly and negatively correlated with social function, in accordance with other follow-up studies and community-based studies [40, 41]. In our study, patients who were likely be the victims of violence participated in social and leisure activities less regularly by using SDS subscales. This may be due to two factors. Fitzgerald et al. reported that those who had no substantial daily activities and showed high degree of psycho-social disability presented high rate of victimization [27]. On the contrary, patients with better social function could get on well with others in regulated environment thus avoiding conflict situations.

This study has several limitations. Firstly, this study was a cross-sectional design, so that the causality between violent victimization and variables cannot be ascertained. Secondly, all participants were in stable condition and conducted in rural primary communities. Therefore, the results cannot be generalized to patients in other illness stages and other settings. Thirdly, some reports demonstrated that women with severe mental illness tend to be victims of sexual violence, which was not showed in this study. In mainland China, sexual violence and harassment is a sensitive topic, it might be interpreted as the lower rate of sexual violence in our sample compared to 20.3% in other studies [42], which affected the capacity of the analyses to confirm the relationships between gender and sexual violence. Lastly, recall bias may have influenced the results.

## Conclusion

In conclusion, individuals with schizophrenia had high risk of violent victimization in rural China and adverse consequences for their social function. We should pay more attention to those patients experiencing violent events, because they simultaneously possible to conduct dangerous behaviors [1, 9, 43]. Urban sample and longitudinal prospective studies will be needed in the future.

## Data Availability

The authors confirm that, for approved reasons, some access restrictions apply to the data underlying the findings. The data set contains identifying participant information, which is not suitable for public deposition. Data might be available upon request by contacting the corresponding author; however, the request must comply with confidentiality and ethics rules of the ethics committee of our institution.

## Abbreviations

BMI: Body Mass Index
BPRS: Brief Psychiatric Rating Scale
DDD: Daily defined dose
FGAs: First-generation antipsychotics
PDD: Prescribed daily dose
QIDS-SR: Quick Inventory of Depressive Symptomatology
SAS: Simpson and Angus Scale of Extrapyradimal Symptoms
SDS: The Sheehan Disability Scale
SGAs: Second--generation antipsychotics
SMI: Severe mental illness
WHOQoL: World Health Organization Quality of Life
